# Supplementary material for: Metabarcoding Analysis of Rhizosphere and Bulk Soils in Bulgaria Reveals Fungal Community Shifts Under Oat–Vetch Intercropping Versus Sole Oat Cultivation
Source: Microorganisms. 2025 Dec 24;14(1):42. doi: 10.3390/microorganisms14010042 (PMC12843743; doi:10.3390/microorganisms14010042)
Supplement: Supplementary file 1 [file microorganisms-14-00042-s001.zip › Supplementary table S2. PERMANOVA comparing fungal community composition.pdf]

Supplementary Table S2. Pairwise PERMANOVA comparing fungal community composition among treatments based on Bray–Curtis dissimilarity. Significant values ($p < 0.05$) are bolded.

Comparison	F-statistic	R^2	*p*-value
Intercrop vs Monocrop Oat	0.00*	0.000	0.001
Intercrop vs Monocrop Vetch	1.234	0.055	0.002
Intercrop vs Green Manure	1.470	0.062	0.001
Monocrop Oat vs Green Manure	1.829	0.078	0.001
Monocrop Vetch vs Green Manure	1.521	0.071	0.003
