# Supplementary figures and images for: Metabarcoding Analysis of Rhizosphere and Bulk Soils in Bulgaria Reveals Fungal Community Shifts Under Oat–Vetch Intercropping Versus Sole Oat Cultivation
Source: Microorganisms. 2025 Dec 24;14(1):42. doi: 10.3390/microorganisms14010042 (PMC12843743; doi:10.3390/microorganisms14010042)

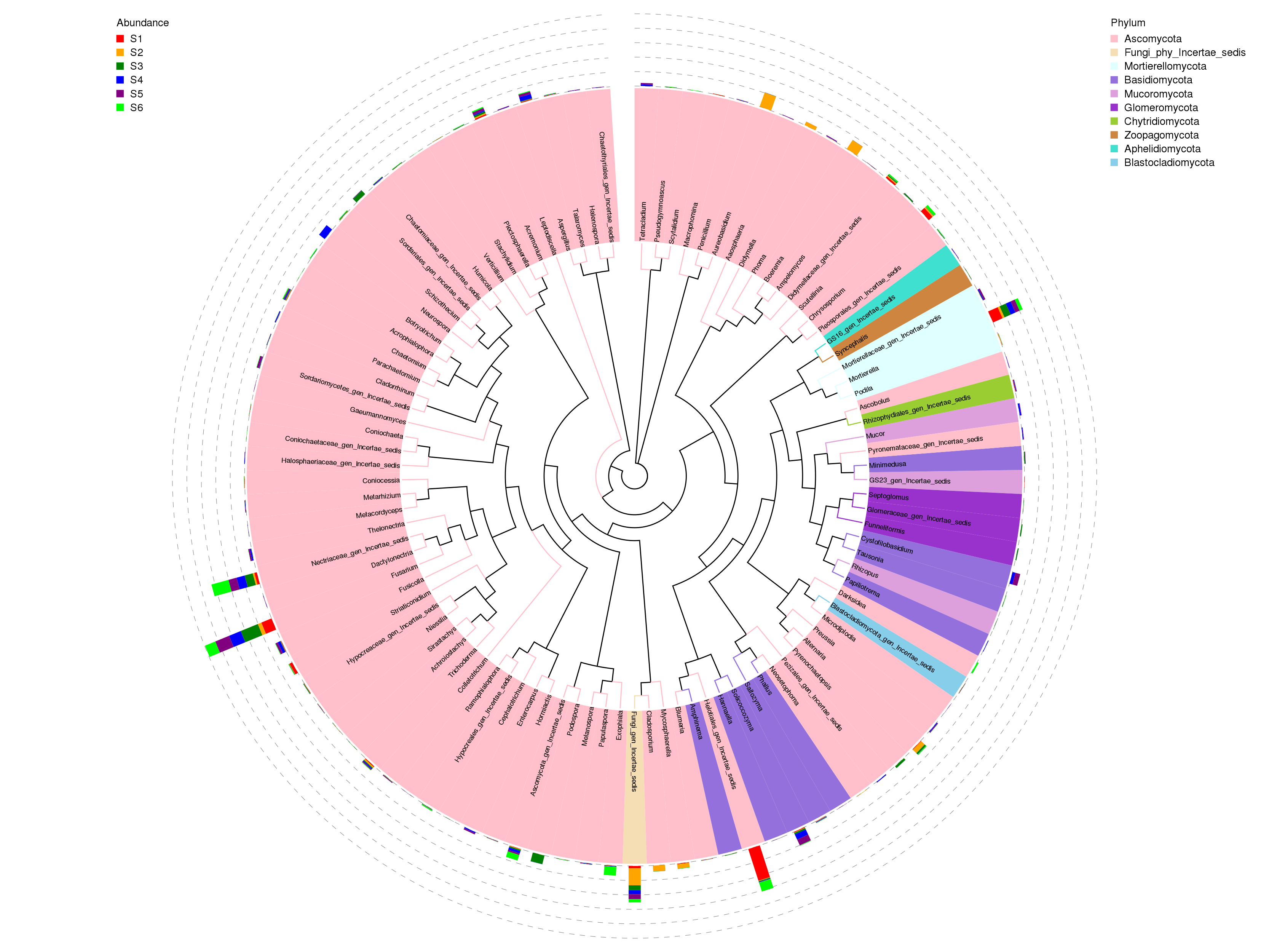

Supplement: Supplementary file 1 [file microorganisms-14-00042-s001.zip › Supplementary figure S1. Phylogenetic relationships and distribution of dominant rhizosphere fungi across soil management treatments..png]

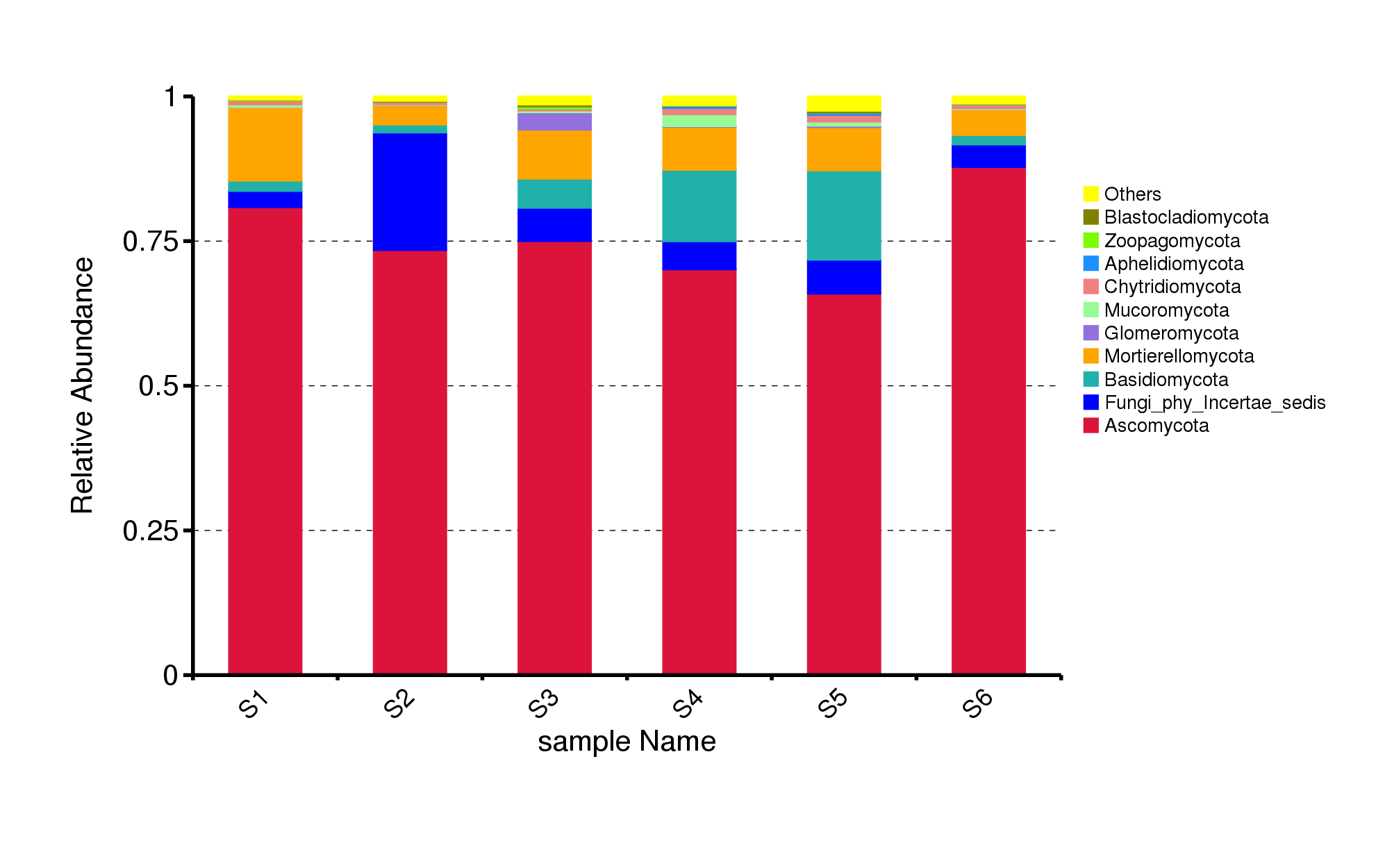

Supplement: Supplementary file 1 [file microorganisms-14-00042-s001.zip › Supplementary figure S2. Relative abundance of fungal phyla in rhizosphere soil samples (S1¿CS6)..png]
